# Earliest radiological progression in glioblastoma by multidisciplinary consensus review

**DOI:** 10.1007/s11060-018-2896-3

**Published:** 2018-05-18

**Authors:** Roelant S. Eijgelaar, Anna M. E. Bruynzeel, Frank J. Lagerwaard, Domenique M. J. Müller, Freek R. Teunissen, Frederik Barkhof, Marcel van Herk, Philip C. De Witt Hamer, Marnix G. Witte

**Affiliations:** 1 Department of Radiation Oncology, The Netherlands Cancer Institute, Amsterdam, The Netherlands; 2 Department of Radiation Oncology, VU University Medical Center, Amsterdam, The Netherlands; 3 Neurosurgical Center Amsterdam, VU University Medical Center, Amsterdam, The Netherlands; 4 Department of Radiology and Nuclear Medicine, VU University Medical Center, Amsterdam, The Netherlands; 5 Institutes of Neurology & Healthcare Engineering, University College London, London, UK; 6 Division of Cancer Sciences, Faculty of Biology, Medicine & Health, University of Manchester and Christie NHS Trust, Manchester, UK

**Keywords:** Glioblastoma, Progression definition, Progression free survival, RANO, MRI

## Abstract

**Background:**

Detection of glioblastoma progression is important for clinical decision-making on cessation or initiation of therapy, for enrollment in clinical trials, and for response measurement in time and location. The RANO-criteria are considered standard for the timing of progression. To evaluate local treatment, we aim to find the most accurate progression location. We determined the differences in progression free survival (PFS) and in tumor volumes at progression (Vprog) by three definitions of progression.

**Methods:**

In a consecutive cohort of 73 patients with newly-diagnosed glioblastoma between 1/1/2012 and 31/12/2013, progression was established according to three definitions. We determined (1) earliest radiological progression (ERP) by retrospective multidisciplinary consensus review using all available imaging and follow-up, (2) clinical practice progression (CPP) from multidisciplinary tumor board conclusions, and (3) progression by the RANO-criteria.

**Results:**

ERP was established in 63 (86%), CPP in 64 (88%), RANO progression in 42 (58%). Of the 63 patients who had died, 37 (59%) did with prior RANO-progression, compared to 57 (90%) for both ERP and CPP. The median overall survival was 15.3 months. The median PFS was 8.8 months for ERP, 9.5 months for CPP, and 11.8 months for RANO. The PFS by ERP was shorter than CPP (HR 0.57, 95% CI 0.38–0.84, p = 0.004) and RANO-progression (HR 0.29, 95% CI 0.19–0.43, p < 0.001). The Vprog were significantly smaller for ERP (median 8.8 mL), than for CPP (17 mL) and RANO (22 mL).

**Conclusion:**

PFS and Vprog vary considerably between progression definitions. Earliest radiological progression by retrospective consensus review should be considered to accurately localize progression and to address confounding of lead time bias in clinical trial enrollment.

**Electronic supplementary material:**

The online version of this article (10.1007/s11060-018-2896-3) contains supplementary material, which is available to authorized users.

## Introduction

Glioblastoma progresses in almost all patients. At progression a standard of care is unavailable and management varies from best supportive care or re-challenge of temozolomide to additional combination treatments or clinical trial participation [1–3]. The definition of progressive disease is ambiguous due to unreliable differentiation between treatment-related imaging phenomena and true disease progression. As a result progressive disease can often only be assumed after substantial growth or occurrence of new distant tumor locations. Detection of glioblastoma progression is important to make decisions on cessation of current treatment or initiation of additional therapy, to enroll patients in clinical trials at comparable stages of advanced disease, and to measure end of treatment response in time and in location. As progression free survival (PFS) has been demonstrated to correlate with overall survival, progression is a useful surrogate endpoint for response assessment of the first treatment in glioblastoma [4].

To standardize the time of progression, the response assessment in neuro-oncology (RANO) working group established radiological criteria for treatment response evaluation and for clinical trial entry, as an update to the MacDonald criteria [5–8]. These RANO criteria are based on standard FLAIR, T2-weighted and T1-weighted MR imaging before and after gadolinium-based contrast agents, which have widely-acknowledged shortcomings to identify pseudoprogression (i.e., treatment-related increase in contrast enhancement without disease progression), and pseudoresponse (i.e. treatment-related decrease of contrast enhancement without disease regression) [9, 10]. In addition, extensive nonenhancing true disease progression can remain unidentified.

In clinical practice, decisions to stop current treatment or start additional therapy for patients with glioblastoma progression are made in multidisciplinary tumor board meetings and can be based on factors other than radiological progression, such as patient condition and motivation, and expectations from additional treatment. This may result in treatment decisions that diverge in timing from progression criteria fulfillment. We considered this as clinical practice progression (CPP).

In this study we focus on the use of progression to evaluate initial local treatment, such as surgery or radiotherapy. For this purpose we postulate earliest radiological progression (ERP) that enables an accurate measurement of the location of first progression. Earliest radiological progression is determined by consensus in a multidisciplinary team that retrospectively reviews all clinical and radiological follow-up information. In this review the development of end stage of disease and its radiological appearance can be taken into account, which could improve the determination of the time of progression and consequently PFS [11].

Here, we quantify the differences in PFS and in tumor volume at the first time of progression using three definitions of progression: ERP, CPP, and RANO criteria in a consecutive patient cohort treated at a tertiary referral center for brain tumors. The aim of this study is to quantify the differences in time to progression and tumor volume based on existing definitions for progression and the novel definition of earliest radiological progression. These definitions are not mutually exclusive and may serve different purposes. Our motivating purpose to compare definitions is to estimate the location of progression within the brain as accurately as possible for treatment evaluation.

## Methods

All patients were identified from an electronic department database, who had newly-diagnosed glioblastoma according to the WHO 2007 classification [12] and were treated in the VU University Medical Center between Jan 1, 2012 and Dec 31, 2013. Brain imaging was retrieved from the hospital PACS. Data closure for radiological follow-up, most recent hospital visit and death status was on March 1, 2016. No patient was lost in follow-up. Time intervals were censored at most recent hospital visit for patients alive at data closure. Informed consent was obtained from all individual participants included in the study.

Of the 97 identified patients, 73 had at least one MRI in follow-up after the postoperative imaging and were therefore included to evaluate progression. The other 24 patients had died early without follow-up MRI, because they had a condition too poor for imaging that would not have had consequences for oncological treatment. Ten patients were still alive at data closure. The median follow-up of patients still alive was 33 months. The patient and treatment characteristics were obtained from the electronic medical records and are listed in Table 1.

**Table 1:** Patient and treatment characteristics

	Study population
Number of patients	73
Median age	62.0
Female	26 (36%)
WHO performance status before treatment	
0	9
1	34
2	26
3	4
Median tumor volume in mL	24
Resection	62 (85%)
Gross total resection	55 (89%)
Median extent of resection	97%
Biopsy	11 (15%)
Chemo-radiotherapy	61 (84%)
With total radiation dose of 60 Gy	53 (87%)
With total radiation dose of 40 Gy	8 (13%)
Radiotherapy only	7 (10%)
Chemotherapy only	4 (5%)
No adjuvant therapy	1 (1%)

Clinical treatment decisions for these patients were made in twice-weekly multidisciplinary tumor board meetings including neurologists, neurosurgeons, neuroradiologists, neuropathologists, radiation- and medical oncologists. The standard of first treatment was surgery in the form of resection or biopsy, followed by chemoradiation consisting of a total dose of 60 Gy conformal radiotherapy in daily fractions of 2 Gy with concomitant temozolomide of 75 mg/m^2^ daily from the first to the last day of radiotherapy, followed by six cycles of adjuvant temozolomide of 150–200 mg/m^2^ for 5 days every 28 days. As we included all newly-diagnosed patients, for some patients treatments were individualized.

Radiological follow-up with MRI was in accordance with national guidelines [13], typically including an immediately postoperative MRI considered as reference, a first follow-up MRI halfway during adjuvant chemotherapy and scheduled MRIs at approximately 3 month intervals after completion of chemotherapy and additional MRIs as necessary based on alterations in patient condition. A standardized MRI protocol included T2-weighted-, FLAIR-, T1-weighted before and after gadolinium-based contrast agents, diffusion-, and perfusion-weighted imaging as previously described in [14].

**Fig. 1:**
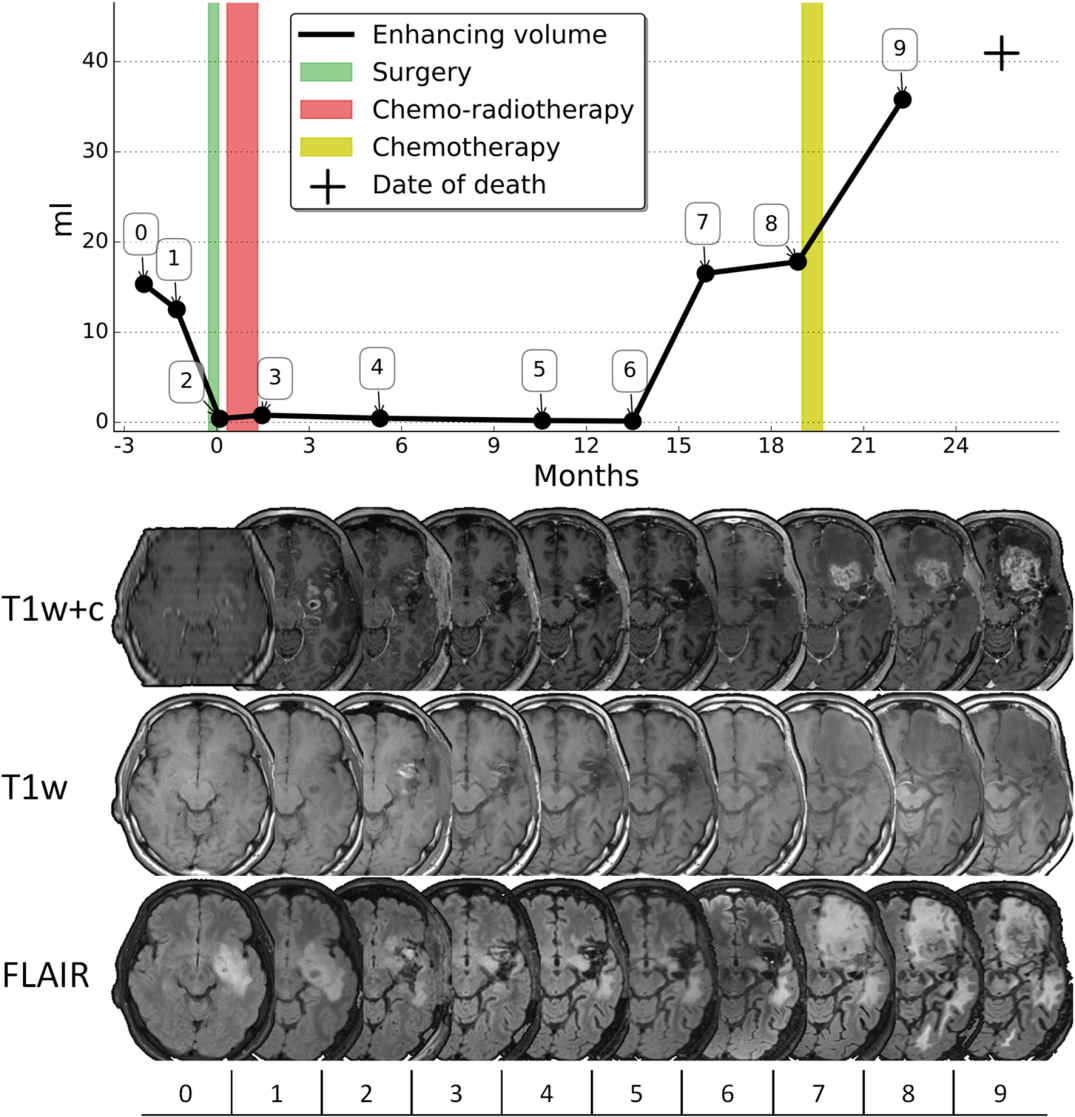
Consensus review progression example. The enhancing tumor volumes and clinical events are plotted in time from diagnosis. Below the graph the corresponding MR images are shown, from top to bottom: the T1-weighted with contrast agent, T1-weighted and FLAIR images. In this case the earliest radiological progression was at the seventh MRI (547 days), the clinical practice progression at the eighth MRI (637 days), and the RANO progression at the ninth MRI (739 days)

Three definitions of first progression were used. First, for ERP, a consensus panel (a neurosurgeon, two radiation oncologists, and a neuroradiologist) re-examined all treatment decisions, the patient’s condition over time and date of death together with all MRIs to trace back the first MRI that demonstrated the radiological progression. Radiological changes were correlated with possible response to additional treatment. In the review process, the scan clearly showing progressive disease was identified and consequently the location of progression was examined on previous scans. ERP was defined as the first scan showing contrast enhancement on the T1-weighted MRI after gadolinium-based contrast agent, at the location that evolved into fatal progressive disease. As the last progression before death was identified, this was considered true progression, thereby excluding pseudoprogression. To facilitate an efficient review a timeline was plotted for each patient with enhancing tumor volumes for every MRI and clinical events, such as oncological treatment, start of dexamethasone, and neurological deterioration (see Fig. 1). MRIs were coregistered for each patient to facilitate review of multiple modalities over time. Second, for CPP, the notes, conclusions and treatment decisions of the multidisciplinary tumor board meetings were reviewed to extract the care team’s interpretation of first progression during care over the course of disease. Third, for RANO progression, the RANO criteria for first progression were applied to follow-up MRIs: a new measurable contrast-enhancing lesion outside of the radiation field or an increase of more than 25% in the sum of the products of largest axial perpendicular diameters of the contrast-enhancing lesion(s) [6].

The number of days was determined between the date of histopathological diagnosis and the date of progression, the date of death—if progression was unobserved, or the most recent hospital visit—if progression was unobserved and the patient was alive at data closure, for all three definitions. For ERP, as progression date we used the date of the MRI, that most accurately represented the origin of first progression; for CPP, the date of the MRI that the tumor board interpreted as first progression; and for RANO, the date of the MRI that fulfilled the criteria.

To determine the tumor volumes a semi-automated volumetric tumor segmentation of the gadolinium-enhanced T1-weighted imaging MRI sequences was used (Brainlab Smartbrush Suite software, BrainLAB AG, Münich, Germany). We considered gadolinium-enhancing tumor and -nonenhancing enclosed necrosis or cyst on T1-weighted images as tumor. Segmentations were performed on the gadolinium-enhanced T1-weighted imaging (generally 1 × 1 × 1 mm voxel size), while taking other modalities into account. The volumetric reconstructions of the segmentations were edited in three orthogonal planes and verified by the expert panel.

For statistical comparison of tumor volumes at progression, we have used the nonparametric Quade test for repeated measures on three groups and the Conover’s Quade post hoc test for group comparisons [15]. For comparison of PFS, we have used Kaplan–Meier survival curve plots and a proportional hazards random effects model to compare the PFS according to the three progression definitions with patients as model frailties with normal distribution for repeated measures [16].

## Results

The 73 patients had on average 3.9 MRIs [interquartile range (IQR) between 2 and 5] in follow-up. The first follow-up MRI after postoperative imaging was obtained at a median of 5.4 months after the date of diagnosis (IQR between 3.2 and 8.8 months).

Not all patients fulfilled all three definitions of progression. ERP was established in 63 (86%) of 73 patients, CPP in 64 (88%), RANO progression in 42 (58%). Of the 63 patients who had died, 57 (90%) did with prior ERP, 57 (90%) with prior CPP, and 37 (59%) with prior RANO progression. Of the 10 patients still alive, six (60%) have had ERP, 7 (70%) CPP, and 5 (50%) RANO progression.

**Fig. 2:**
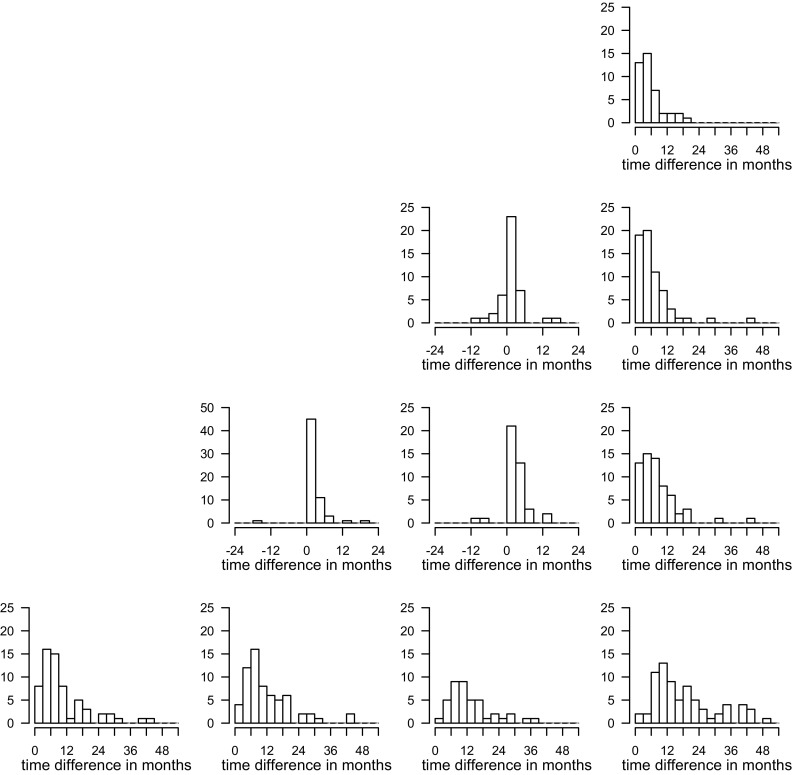
Histograms of time differences in months between diagnosis, progression according to earliest radiological detection, clinical practice, and RANO criteria, and death. The time difference is from the event mentioned on the left to the event at the bottom

The time differences between diagnosis, progressions, and death are plotted in Fig. 2. Some noticeable differences were observed. In one patient CPP was 16 months before the ERP, because the consensus panel considered the CPP false-positive as the presumed progression remained stable for 16 months without treatment. In two other patients the RANO progression was 8 and 11 months before EPR, because the consensus panel considered the RANO progression false-positive based on spontaneous regression of the presumed progression. The 26 (41%) patients who died without prior RANO progression can be considered false-negative RANO progressions. The 6 (10%) patients who died without prior CPP can be considered false-negative clinical decisions.

**Fig. 3:**
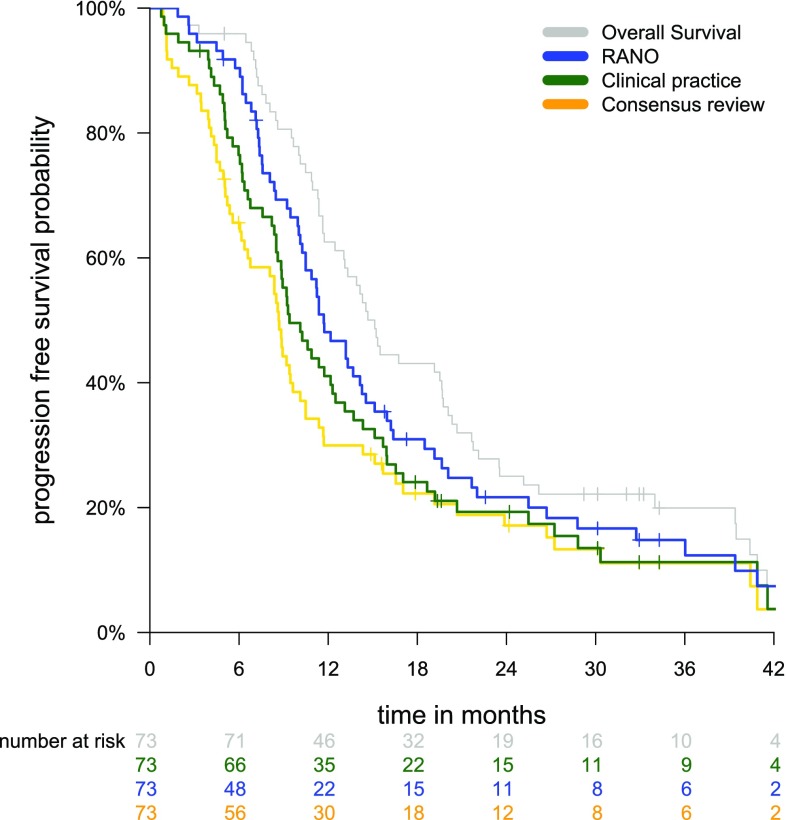
Progression free survival Kaplan–Meier curves for the definitions according to earliest radiological progression (orange), clinical practice progression (green), and RANO criteria (blue). As a reference, the overall survival curve is plotted in grey

The PFS curves according to the three definitions and the overall survival are plotted in Fig. 3. As a reference, the median overall survival was 15.3 months and the percentage of patients alive at 12 months was 63%. The median PFS was 8.8 months until ERP, 9.5 months until CPP, and 11.8 months until RANO progression. The percentage of patients with PFS at 12 months was 29% for ERP, 40% for CPP, and 47% for RANO progression. PFS until ERP was shorter than until CPP [Hazard ratio (HR) for progression 0.57, 95% CI 0.38–0.84, p = 0.004], and shorter than until RANO progression (HR 0.29, 95% CI 0.19–0.43, p < 0.001). PFS until RANO progression was longer than until CPP (HR 1.75, 95% CI 1.19–2.58, p = 0.004). In a subgroup analysis with 54 patients who had a gross total resection and chemoradiation, similar differences in PFS were observed (Supplementary Fig. 1).

**Fig. 4:**
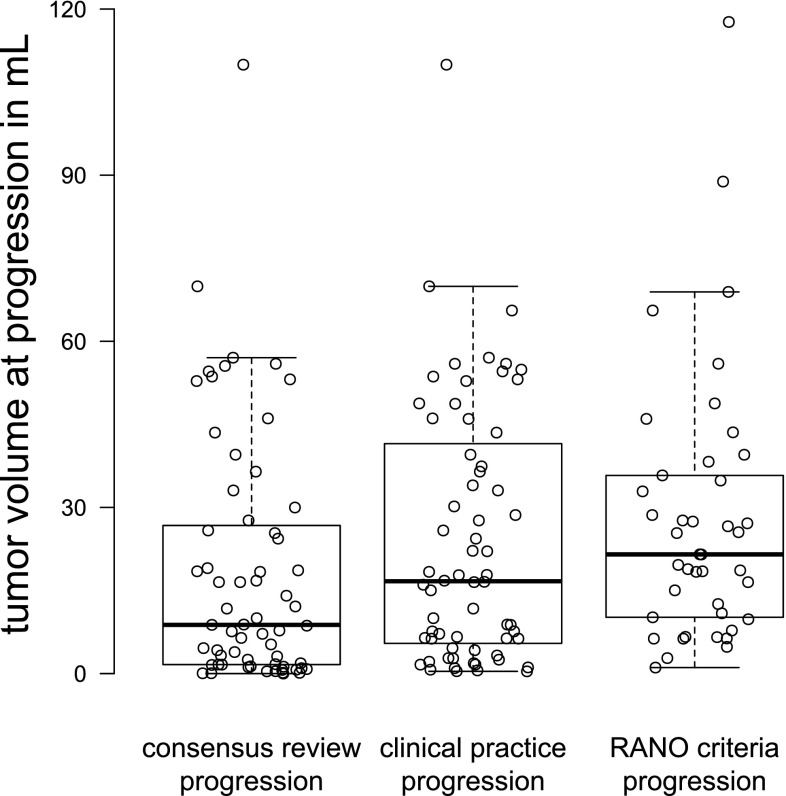
Box plot of tumor volumes at progression according to progression definitions of clinical practice, RANO criteria, and consensus review

The tumor volumes at progression were different among the three definitions (Quade F = 16.9, df = 2, p < 0.0001) as shown in Fig. 4. The smallest tumor volumes were observed at ERP with a median of 8.8 mL (IQR 1.6–27 mL), followed by volumes at CPP with a median of 17 mL (IQR 5.5–42 mL) and at RANO progression with a median of 22 mL (IQR 10–36 mL). The median difference in tumor volume between ERP and CPP was 0 mL (IQR 0–5 mL) and between ERP and RANO was 10 mL (IQR 3.5–27 mL).

## Discussion

In this study we postulate earliest radiological progression as comprehensive definition of glioblastoma progression by consensus of a multidisciplinary panel, that is only available after the course of disease using all available imaging, treatment decisions, neurological deterioration and ultimately death from disease. We demonstrate that the PFS using earliest radiological progression is shorter and detects smaller tumor volume than the tumor board meeting’s interpretation of progression, and even more so than the RANO criteria for progressive disease.

The unambiguous assessment of glioblastoma progression on MRI is notoriously difficult for several reasons. First, standard MRI sequences do not adequately reflect the extent of glioblastoma infiltration with T2-weighted imaging being too sensitive for tumor detection and gadolinium-enhanced T1-weighted imaging not being specific enough [17]. Second, treatment can induce MRI abnormalities, that mimick glioblastoma progression, such as resulting from surgery [18], radiotherapy [19], antiangiogenetic agents [20], and immunotherapy [21]. Third, the correlation between patient condition and MRI abnormalities is not strong [22, 23]. Fourth, MRI monitoring is necessarily discontinuous, and the level of progression detection is as fine-grained as the scheduled time intervals between MRIs. In clinical practice this often results in postponed treatment decisions until additional MRI followup is obtained at shortened intervals to accumulate arguments for further radiological progression.

To improve the distinction of glioblastoma progression and treatment-related MRI abnormalities, advanced imaging techniques have been introduced [24–27], such as MR perfusion-weighted imaging [28, 29], MR diffusion-weighted imaging [30, 31], MR spectroscopy [32, 33], and Positron Emission Tomography [34, 35]. A multimodal combination of imaging techniques has been reported to be more accurate than single imaging modalities [36–38]. Another strategy is to determine progressive disease from routinely collected healthcare data on treatments and readmission without imaging criteria [3]. Although promising, these strategies have so far not been incorporated in standard care.

Clinical decisions were made earlier in our data than the RANO criteria were fulfilled. Several reports discussing the RANO criteria have mentioned that these criteria are not customarily used as standard in clinical practice [39–41]. In a recent survey among radiologists in Europe only 27% obtained measurements according to RANO criteria [42]. Kazda et al. identified a difference in time to progression between clinical decisions and RANO criteria in anaplastic astrocytoma [43]. No publications were identified that have reported on this comparison in glioblastoma.

Strengths of this study include a long term and complete followup of this patient cohort. Furthermore, notes and conclusions from tumor board meetings were well-documented to evaluate the progression definition of clinical practice. Finally a custom MRI viewing setup facilitated efficient imaging review for the multidisciplinary consensus review. Limitations of this study are that this was a single center study with clinical practice interpretation and consensus review by one multidisciplinary team. The interpretations in clinical practice and follow-up frequency may vary between centers. As a result the validity of extrapolation of our results to other teams and centers remains uncertain and should be investigated in future research.

The implications from our findings can be several. The earliest radiological progression should be considered to evaluate the relation between the location of progression and initial local therapy, such as radiotherapy or surgery. It can also be of added value for clinical trial inclusion by allowing correction of lead time bias and confounding due to tumor growth, inherent to the RANO criteria for trial enrollment and response evaluation. Furthermore, it can be useful for evaluation of the quality of clinical decision-making by multidisciplinary teams. The earliest radiological progression can only be determined at late advanced stages of disease and is therefore not a suitable alternative for routine care or for clinical trial enrollment. The minimum requirements for expertise of a multidisciplinary panel, the reproducibility and the optimal scheduled time interval between follow-up MRIs for determination of earliest radiological progression are undetermined.

## Electronic supplementary material

Below is the link to the electronic supplementary material.

Supplementary Figure 1. Progression free survival Kaplan–Meier curves for the subgroup of 54 patients with gross total resection and chemoradiotherapy. The color coding is identical to Figure 3. Supplementary material 1 (EPS 52 KB)

## References

[1] Seystahl K, Wick W, Weller M (2016). Therapeutic options in recurrent glioblastoma: an update. Crit Rev Oncol/Hematol.

[2] Tosoni A, Franceschi E, Poggi R, Brandes AA (2016). Relapsed glioblastoma: treatment strategies for initial and subsequent recurrences. Curr Treat Options Oncol.

[3] Kelly C, Majewska P, Ioannidis S, Raza MH, Williams M (2017). Estimating progression-free survival in patients with glioblastoma using routinely collected data. J Neuro-oncol.

[4] Han K, Ren M, Wick W, Abrey L, Das A, Jin J (2014). Progression-free survival as a surrogate endpoint for overall survival in glioblastoma: a literature-based meta-analysis from 91 trials. Neuro-oncology.

[5] Macdonald DR, Cascino TL, Schold SC, Cairncross JG (1990). Response criteria for phase II studies of supratentorial malignant glioma. J Clin Oncol.

[6] Wen PY, Macdonald DR, Reardon DA, Cloughesy TF, Sorensen AG, Galanis E (2010). Updated response assessment criteria for high-grade gliomas: response assessment in neuro-oncology working group. J Clin Oncol.

[7] Chang SM, Wen PY, Vogelbaum MA, Macdonald DR, van den Bent MJ (2015). Response assessment in neuro-oncology (RANO): more than imaging criteria for malignant glioma: table 1. Neuro-oncol Pract.

[8] Huang RY, Rahman R, Ballman KV, Felten SJ, Anderson SK, Ellingson BM (2016). The impact of T2/FLAIR evaluation per RANO criteria on response assessment of recurrent glioblastoma patients treated with bevacizumab. Clin Cancer Res.

[9] Wick W, Chinot OL, Bendszus M, Mason W, Henriksson R, Saran F (2016). Evaluation of pseudoprogression rates and tumor progression patterns in a phase III trial of bevacizumab plus radiotherapy/temozolomide for newly diagnosed glioblastoma. Neuro-oncology.

[10] Ellingson BM, Chung C, Pope WB, Boxerman JL, Kaufmann TJ (2017). Pseudoprogression, radionecrosis, inflammation or true tumor progression? Challenges associated with glioblastoma response assessment in an evolving therapeutic landscape. J Neurooncol.

[11] Vogelbaum M, Jost S, Aghi MK, Heimberger AB, Sampson JH, Wen PY (2012). Application of novel response/progression measures for surgically delivered therapies for gliomas: response assessment in neuro-oncology (RANO) working group. Neurosurgery.

[12] Louis DN, Ohgaki H, Wiestler OD, Cavenee WK, Burger PC, Jouvet A (2007). The 2007 WHO classification of tumours of the central nervous system. Acta Neuropathol.

[13] Netherlands Comprehensive Cancer Organisation (IKNL) (2007) Cancer clinical practice guidelines, Neuro-oncology, Gliomas, version 2.0

[14] Belhawi SMK, Hoefnagels FWA, Baaijen JC, Sanchez Aliaga E, Reijneveld JC, Heimans JJ (2011). Early postoperative mri overestimates residual tumour after resection of gliomas with no or minimal enhancement. Eur Radiol.

[15] Quade D (1979) Using weighted rankings in the analysis of complete blocks with additive block effects. J Am Stat Assoc 74(367):680. http://www.jstor.org/stable/2286991?origin=crossref

[16] O’Quigley J, Stare J (2002). Proportional hazards models with frailties and random effects. Stat Med.

[17] Verburg N, Hoefnagels FWA, Barkhof F, Boellaard R, Goldman S, Guo J (2017). Diagnostic accuracy of neuroimaging to delineate diffuse gliomas within the brain: a meta-analysis. AJNR Am J Neuroradiol.

[18] Ulmer S, Braga TA, Barker FG, Lev MH, Gonzalez RG, Henson JW (2006). Clinical and radiographic features of peritumoral infarction following resection of glioblastoma. Neurology.

[19] Brandsma D, Stalpers L, Taal W, Sminia P, van den Bent MJ (2008). Clinical features, mechanisms, and management of pseudoprogression in malignant gliomas. Lancet Oncol.

[20] Iwamoto FM, Abrey LE, Beal K, Gutin PH, Rosenblum MK, Reuter VE (2009). Patterns of relapse and prognosis after bevacizumab failure in recurrent glioblastoma. Neurology.

[21] Okada H, Weller M, Huang R, Finocchiaro G, Gilbert MR, Wick W (2015). Immunotherapy response assessment in neuro-oncology: a report of the RANO working group. Lancet Oncol.

[22] Meyers CA, Hess KR (2003). Multifaceted end points in brain tumor clinical trials: cognitive deterioration precedes MRI progression. Neuro-oncology.

[23] Armstrong TS, Vera-Bolanos E, Gning I, Acquaye A, Gilbert MR, Cleeland C (2011). The impact of symptom interference using the MD anderson symptom inventory-brain tumor module (MDASI-BT) on prediction of recurrence in primary brain tumor patients. Cancer.

[24] Shah AH, Snelling B, Bregy A, Patel PR, Tememe D, Bhatia R (2013). Discriminating radiation necrosis from tumor progression in gliomas: a systematic review what is the best imaging modality?. J Neuro-oncol.

[25] Ryken TC, Aygun N, Morris J, Schweizer M, Nair R, Spracklen C (2014). The role of imaging in the management of progressive glioblastoma: a systematic review and evidence-based clinical practice guideline. J Neuro-oncol.

[26] Shiroishi MS, Boxerman JL, Pope WB (2016). Physiologic MRI for assessment of response to therapy and prognosis in glioblastoma. Neuro-oncology.

[27] Huang RY, Neagu MR, Reardon DA, Wen PY (2015). Pitfalls in the neuroimaging of glioblastoma in the era of antiangiogenic and immuno/targeted therapy—detecting illusive disease, defining response. Front Neurol.

[28] Barajas RF, Chang JS, Segal MR, Parsa AT, McDermott MW, Berger MS (2009). Differentiation of recurrent glioblastoma multiforme from radiation necrosis after external beam radiation therapy with dynamic susceptibility-weighted contrast-enhanced perfusion MR imaging. Radiology.

[29] Gasparetto EL, Pawlak MA, Patel SH, Huse J, Woo JH, Krejza J (2009). Posttreatment recurrence of malignant brain neoplasm: accuracy of relative cerebral blood volume fraction in discriminating low from high malignant histologic volume fraction. Radiology.

[30] Lee WJ, Choi SH, Park C-K, Yi KS, Kim TM, Lee S-H (2012). Diffusion-weighted MR imaging for the differentiation of true progression from pseudoprogression following concomitant radiotherapy with temozolomide in patients with newly diagnosed high-grade gliomas. Acad Radiol.

[31] Chu HH, Choi SH, Ryoo I, Kim SC, Yeom J, Shin H (2013). Differentiation of true progression from pseudoprogression in glioblastoma treated with radiation therapy and concomitant temozolomide: comparison study of standard and high-b-value diffusion-weighted imaging. Radiology.

[32] Sawlani V, Taylor R, Rowley K, Redfern R, Martin J, Poptani H (2012). Magnetic resonance spectroscopy for differentiating pseudo-progression from true progression in GBM on concurrent chemoradiotherapy. Neuroradiol J.

[33] Kazda T, Bulik M, Pospisil P, Lakomy R, Smrcka M, Slampa P (2016). Advanced MRI increases the diagnostic accuracy of recurrent glioblastoma: single institution thresholds and validation of MR spectroscopy and diffusion weighted MR imaging. NeuroImage Clin.

[34] Kebir S, Fimmers R, Galldiks N, Schäfer N, Mack F, Schaub C (2016). Late pseudoprogression in glioblastoma: diagnostic value of dynamic O-(2-[18F]fluoroethyl)-l-tyrosine PET. Clin Cancer Res.

[35] Galldiks N, Dunkl V, Stoffels G, Hutterer M, Rapp M, Sabel M (2015). Diagnosis of pseudoprogression in patients with glioblastoma using O-(2-[18F]fluoroethyl)-l-tyrosine PET. Eur J Nucl Med Mol Imaging.

[36] Boonzaier NR, Larkin TJ, Matys T, van der Hoorn A, Yan J-L, Price SJ (2017). Multiparametric MR imaging of diffusion and perfusion in contrast-enhancing and nonenhancing components in patients with glioblastoma. Radiology.

[37] Park JE, Kim HS, Goh MJ, Kim SJ, Kim JH (2015). Pseudoprogression in patients with glioblastoma: assessment by using volume-weighted voxel-based multiparametric clustering of MR imaging data in an independent test set. Radiology.

[38] Kim HS, Goh MJ, Kim N, Choi CG, Kim SJ, Kim JH (2014). Which combination of MR imaging modalities is best for predicting recurrent glioblastoma? Study of diagnostic accuracy and reproducibility. Radiology.

[39] Yang D (2016). Standardized mri assessment of high-grade glioma response: a review of the essential elements and pitfalls of the rano criteria. Neuro-oncol Pract.

[40] Huber G, Alber G, Bette S, Kaesmacher J (2017). Progressive disease in glioblastoma: benefits and limitations of semi-automated volumetry. PLoS ONE.

[41] Pope W, Hessel C (2011) Response assessment in neuro-oncology criteria: implementation challenges in multicenter neuro-oncology trials. Am J Neuroradiol 32(5):794–797. http://www.ajnr.org/content/32/5/794 10.3174/ajnr.A2582 PMC7965568 21474628

[42] Thust SC, Heiland S, Falini A, Jäger HR, Waldman AD, Sundgren PC (2018). Glioma imaging in europe: a survey of 220 centres and recommendations for best clinical practice. Eur Radiol.

[43] Kazda T, Hardie JG, Pafundi DH, Kaufmann TJ, Brinkmann DH, Laack NN (2015). Evaluation of rano response criteria compared to clinician evaluation in who grade iii anaplastic astrocytoma: Implications for clinical trial reporting and patterns of failure. J Neuro-oncol.

